# Epinephrine drives human M2a allergic macrophages to a regulatory phenotype reducing mast cell degranulation in vitro

**DOI:** 10.1111/all.14299

**Published:** 2020-04-17

**Authors:** Jelena Gotovina, Rodolfo Bianchini, Judit Fazekas‐Singer, Ina Herrmann, Giulia Pellizzari, Ian D. Haidl, Karin Hufnagl, Sophia N. Karagiannis, Jean S. Marshall, Erika Jensen‐Jarolim

**Affiliations:** ^1^ Comparative Medicine The Interuniversity Messerli Research Institute of the University of Veterinary Medicine Vienna Medical University of Vienna and University of Vienna Vienna Austria; ^2^ Institute of Pathophysiology and Allergy Research Center of Pathophysiology, Infectiology and Immunology Medical University of Vienna Vienna Austria; ^3^ Department for Companion Animals and Horses Small Animal Clinic Internal Medicine University of Veterinary Medicine Vienna Austria; ^4^ St John's Institute of Dermatology School of Basic and Medical Biosciences Guy's Cancer Centre King's College London London UK; ^5^ Department of Microbiology and Immunology Dalhousie University Halifax NS Canada; ^6^ Breast Cancer Now Unit School of Cancer and Pharmaceutical Sciences Guy's Cancer Centre King's College London London UK

**Keywords:** allergy, beta2‐adrenergic receptor, epinephrine, M2a macrophages, mast cell

To the Editor,

As the prevalence of allergies rises, the impact of social factors such as physiological stress has gained much attention. While stress is suggested to exacerbate allergic conditions, including asthma and atopic dermatitis, less is known about the effect of acute stress mediator epinephrine on allergic M2a macrophages in a Th2 environment. This study aimed to investigate whether human M2a macrophages express adrenergic receptors to respond to epinephrine and what effect epinephrine could exhibit on M2a macrophages in an in vitro Th2 environment. We further assessed whether epinephrine‐treated M2a macrophages could affect IgE‐mediated degranulation in human mast cells in vitro*.*

To study the effect of epinephrine on human M2a macrophages*,* we isolated monocytes from healthy donors and matured them in the presence of M‐CSF according to a standard protocol^1^ into monocyte‐derived macrophages (M0). M0 were subsequently treated with IL‐4 and IL‐13 to differentiate them into M2a phenotype, which showed higher expression of CD206 marker and IL‐10 production. Detailed information on this study is available in this article's online supplementary information. The presence of the β2‐adrenergic receptor (β2‐AR) was confirmed in the M2a subtype, but no expression of α2A‐AR, β1‐AR, and β3‐AR was detected (online repository; Figure S2D‐E). The 16‐hour treatment of M2a macrophages with 1 µmol/L epinephrine led to a significant upregulation of the cytokines IL‐10 (*P* = .0131), TNF (*P* = .0012) and IL‐6 (*P* = .0001), while no M1 marker IL‐12 was detected (Figure 1A‐D). This effect was not observed in the supernatants of M2a macrophages treated with the vehicle (negative control). Also, CD86 surface marker expression was significantly upregulated (*P* = .0313) (Figure 1G, Figure S3), indicating an antigen presentation capacity of this phenotype. Since epinephrine can induce cytokine production already after a few hours, we also observed the mRNA production of IL‐10, IL‐6, TNF, IL‐1β , and CCL1 after 2 hours. Other M2 markers, including CCL2, CCL22, CCL18, and TGF‐β, were less affected, and expression of IFN‐γ was not detected after epinephrine treatment (Figure 2A). The production of anti‐inflammatory IL‐10 cytokine alongside IL‐6, TNF, and IL‐1β and upregulation of CD86 suggest that epinephrine can drive M2a macrophages towards an immunoregulatory M2b phenotype in vitro. Since the M2b phenotype is commonly induced by exposure to immune complexes and TLR ligands, which was not the case in our study, and we did not observe CCL1 production in the supernatants of epinephrine‐treated M2a macrophages,^1^, ^2^ we termed this immunoregulatory phenotype “M2b‐like.” It is important to note that the immunoregulatory function of this phenotype was confirmed in vitro on human cord blood–derived mast cells (CBMCs), where treatment with supernatants from epinephrine‐treated M2b‐like macrophages significantly reduced the IgE‐mediated β‐hexosaminidase degranulation (*P* = .0013). Interestingly, this effect was significantly pronounced compared to treatment with epinephrine alone (*P* ≤ .05) (Figure 2B).

**FIGURE 1:**
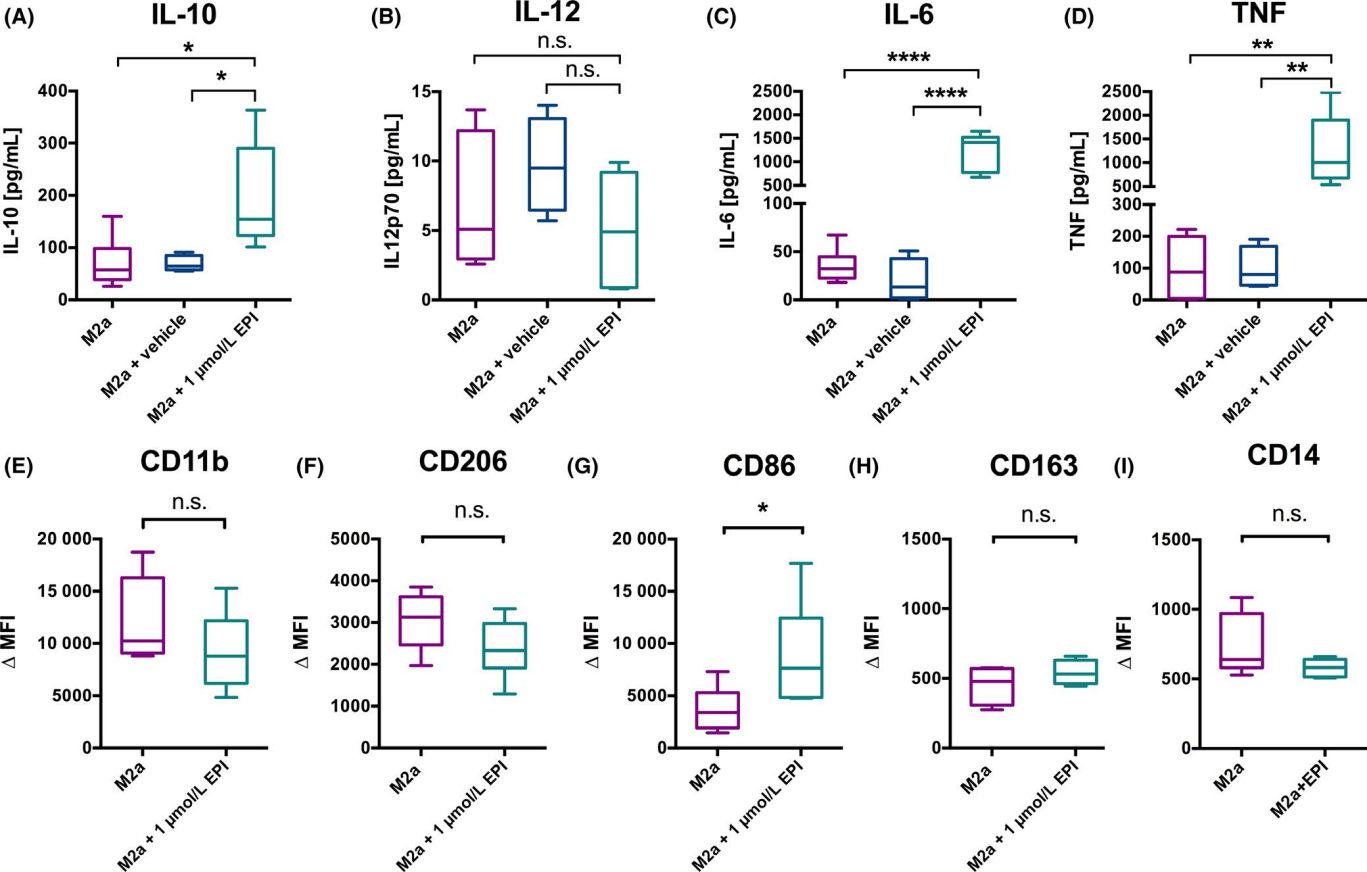
Epinephrine effect on M2a cytokine production assessed by ELISA and surface marker expression assessed by flow cytometry. M2a macrophages (purple) were incubated overnight (16 h) with 1 µmol/L epinephrine (EPI) (teal) or vehicle (blue). IL‐10 (A), IL‐12 p70 (B), IL‐6 (C) and TNF (D) cytokines were assessed in supernatants (mean ± SD of six independent donors), and CD11b (E), CD206 (F), CD86 (G), CD163 (H) and CD14 (I) surface expression (mean ± SD of six independent donors) was assessed on M2a macrophages (purple) or EPI‐treated M2a (teal). ∆MFI is calculated after isotype control MFI subtraction

**FIGURE 2:**
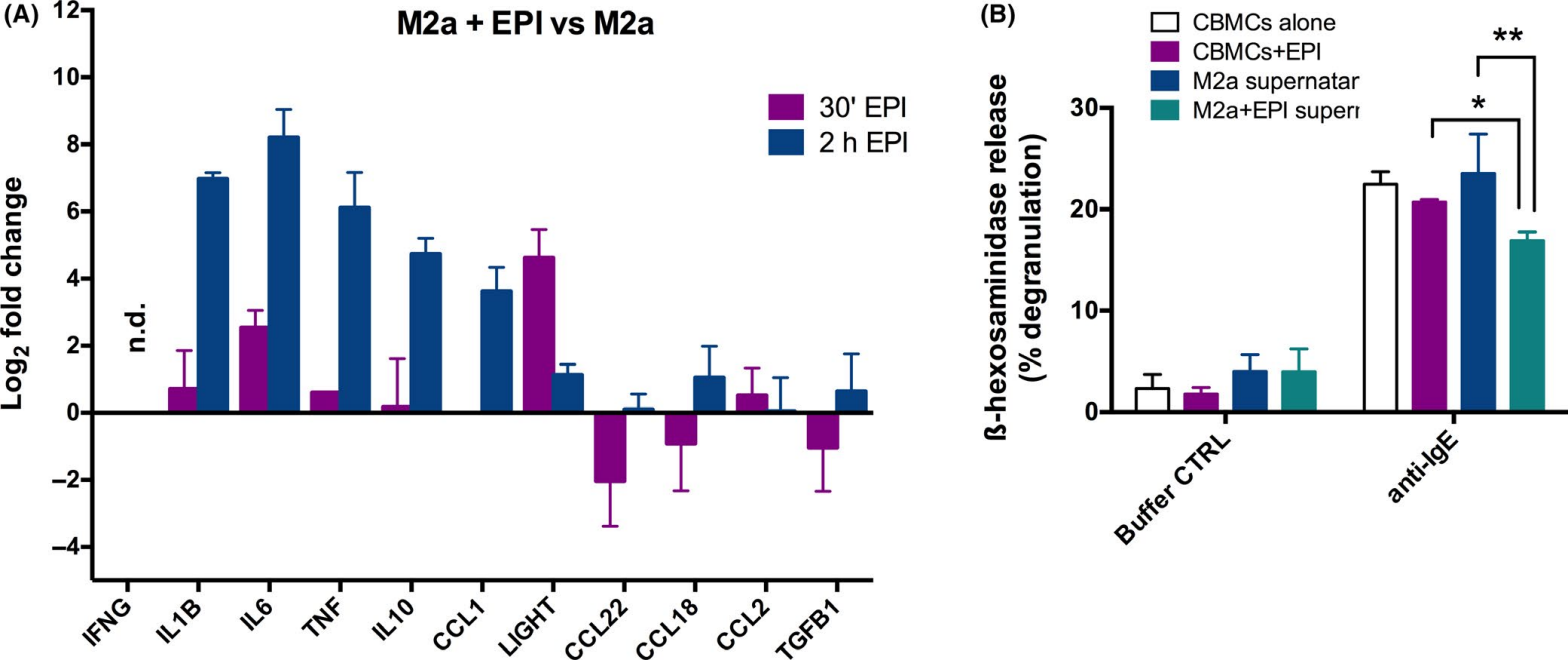
Transcriptional profiling of epinephrine‐treated vs untreated M2a macrophage genes (A) and FcεRI‐mediated β‐hexosaminidase release in CBMCs. (A) Incubation with 1 µmol/L epinephrine (EPI) for 30 min (purple bars) and 2 h (blue bars) (mean ± SD of three independent donors). (B) FcεRI‐mediated β‐hexosaminidase release assessed in CBMCs after overnight incubation with supernatants from M2a macrophages (blue bars), epinephrine‐treated M2a macrophages (teal bars) or 1 µmol/L epinephrine (purple bars) (two different CBMC batches and three different PBMC donors (n = 6))

To the best of our knowledge, this is the first report about the presence of the β2‐AR receptor on the M2a macrophage phenotype, which is an important player in allergy. We, however, acknowledge that our study has its limitations. Although µM epinephrine in mouse cells can induce regulatory macrophages^3^ and dose‐dependent studies of epinephrine on human monocytes revealed the strongest effect on chemokine/cytokine production in 1‐10 µmol/L concentration range, often used to stimulate human monocytes in vitro,^4^, ^5^ our results do not necessarily translate into real human setting. However, there is reason to believe that during stress the local epinephrine concentrations at the immunological synapse are higher than in circulation due to sympathetic neuronal discharge and local catecholamine production from neighbouring immune cells (previously termed “diffusely expressed adrenergic organ”^6^). Another limitation of results (Figure 2A) is the normalization against a single housekeeping gene. We acknowledge that under given conditions, using a second gene for normalization had been advisable. This was a study on epinephrine effect on in vitro Th2 inflammation. To translate these data and develop targeted therapies in the future, it would be important to obtain the information on the exact signalling pathway that epinephrine might have activated on M2a human macrophages and drive the M2b‐like phenotype. FcR signalling known to induce the M2b phenotype by activating phosphoinositide 3‐kinase (PI3K)^2^ , may be a possible pathway induced by epinephrine in our study; furthermore, catecholamine activation of a β2‐AR noncanonical pathway through phosphoinositol 3‐kinase (PI3K) induced regulatory macrophages in mice.^3^

Even though M2b‐like macrophages retain the ability to produce many pro‐inflammatory cytokines including IL‐6, TNF, and IL‐1β, the upregulation of IL‐10 (IL‐10^high^/IL‐12^low^) is certainly a central part of this phenotype and in the range reported in the previous studies.^1^, ^7^ Future studies should address the involvement of IL‐10, but also IL‐6, TNF, and IL‐1β in the observed reduction of β‐hexosaminidase production by CBMCs, as this was beyond the scope of this work. However, IL‐10 could be a possible target, since it was shown to suppress the FcεRI signalling pathway and reduce histamine release in CBMCs^8^ or to directly affect the FcεRI expression and reduce degranulation in human skin mast cells.^9^ Although mast cells are known to express β2‐AR and can respond to epinephrine stimulation (control treatment; Figure 2B), the observed effect on degranulation of CBMCs with supernatants from M2b‐like macrophages was significantly higher than the impact of epinephrine alone. Due to its short half‐life and its instability under supernatant storage conditions (−20°C), epinephrine is not expected to be present in the supernatants of M2b‐like macrophages.

In conclusion, the treatment of human allergic M2a macrophages with epinephrine led to a phenotypic switch to a macrophage subtype, which we term “M2b‐like.” In vitro data suggest that the M2b‐like phenotype suppresses the IgE‐dependent release of inflammatory mediators from mast cells. In allergic patients, acute stress may drive the plasticity of macrophages towards a regulatory M2b phenotype and reduce allergic symptoms, but further studies are needed to translate the results of this in vitro study into real life. However, as recently demonstrated in a clinical study in which the effects of acute stress on skin prick testing greatly varied among individuals,^10^ the net outcome of short‐term acute stress in patients seems to be more complex and also depends on coping mechanisms. Together, our findings support further studies on the role of acute stress mediators in allergies.

## CONFLICTS OF INTEREST

Dr Gotovina, Dr Bianchini, Dr Singer, Dr Herrmann, Dr Pellizzari, Dr Haidl, Dr Hufnagl and Dr Marshall have nothing to disclose. Dr Karagiannis reports grants from NIHR Biomedical Research Centre at Guy's and St Thomas's Hospitals NHS Trust and King's College London; Medical Research Council; Breast Cancer Now; CR UK/NIHR in England/DoH for Scotland, Wales and Northern Ireland Experimental Cancer Medicine Centre; Cancer Research UK; Guy's and St Thomas's Charity, during the conduct of the study; and IGEM Therapeutics Ltd., outside the submitted work; in addition, Dr Karagiannis has a patent (IgE antibody technology) issued to IGEM Therapeutics Ltd. Dr Jensen‐Jarolim reports grants and other from Biomedical International R + D GmbH, Vienna, and Bencard Allergie GmbH, Germany, and other from Allergy Therapeutics Ltd., UK, outside the submitted work.

## Funding information

This study was supported by the Austrian Science Fund grants W1205‐B09 (CCHD) and SFB F4606‐B28 to EJJ. The authors acknowledge support by the Medical Research Council (MR/L023091/1) (SNK); the Academy of Medical Sciences (SNK); CR UK//NIHR in England/DoH for Scotland, Wales and Northern Ireland Experimental Cancer Medicine Centre (C10355/A15587) (SNK); Cancer Research UK (C30122/A11527; C30122/A15774) (SNK, GP); and Breast Cancer Now (147), working in partnership with Walk the Walk (SNK). The research was supported by the National Institute for Health Research (NIHR) Biomedical Research Centre (BRC) based at Guy's and St. Thomas' NHS Foundation Trust and King's College London (IS‐BRC‐1215‐20006) (SNK).

## Supporting information

Supplementary Material
