# Supplementary material for: Epinephrine drives human M2a allergic macrophages to a regulatory phenotype reducing mast cell degranulation in vitro
Source: Allergy. 2020 Apr 17;75(11):2939–42. doi: 10.1111/all.14299 (PMC7687235; doi:10.1111/all.14299)
Supplement: Supplementary file 1 — Supplementary Material [file ALL-75-2939-s001.docx]

**Online Repository Material**

**Supplementary results**

**MDMs purity**

As reported in the previous study^E1^, the purity of adherent monocyte-derived macrophages (CD3^-^CD11b^+^CD86^+^) after 72h in M-CSF-rich medium used in the experiments was >85±5% (**Fig. S1 a-f**). The majority of MDM contamination originates from CD3+ cells (45-70%^E2^), which are reported in our study to be 3% on average. Taking into consideration the frequency of total B cells within PBMCs of 5-15% (approx. 1/5 of CD3+ cells) and NK cells of 5-10% (approx. 1/7 of CD3+ cells^E2^), we can assume that the total lymphocyte contamination in our study is < 5%.

**FcεRI expression on CBMCs**

Enhancement of FcεRI expression on CBMCs cultured for 4 days in the presence of rhIL-4 and human myeloma IgE was confirmed by flow cytometry, as previously reported^E3^ (**Fig. S1 g**).

**Phenotypic characterization of M2a macrophages**

M2a macrophages produce IL-10 cytokine and show high expression of CD206 surface marker^E4^. M2a phenotype further showed expression of CD11b pan-macrophage marker^E5^ as well as higher CD86 expression. We also observed lower expression of CD14 marker previously reported in M2a macrophages differentiated from MDMs cultured in XVivo medium^E6^ (**Fig. S2 a-c**).

**β2-AR expression on M2a macrophages**

M2a macrophages showed the expression of β2-AR both in RT-PCR and immunofluorescence staining (**Fig. S2 d-e**). We did not observe the mRNA expression of α2A-AR, β1-AR and β3-AR on human M2a macrophages (Fig. S2 d).

**Materials and Methods**

*Human primary cells and antibodies***Human primary cells: MDMs:** PBMCs were isolated using Ficoll-Paque Plus (GE Healthcare Life Sciences, South Logan, UT) from ten healthy human donors (ICF obtained and the study approved by EC of the Medical University of Vienna (ECS2007/2016) and 2x10^6^cells/ml seeded into 6-well plates (Falcon, Corning, NY). Non-adherent cells were washed away after 2h with HBSS (GE Healthcare Life Sciences, SouthLogan, UT) and adherent cells (monocytes) kept in RPMI 1640 medium (Sigma-Aldrich, Saint Louis, MO) supplemented with 10% heat-inactivated FBS (Thermo Fisher Scientific, Waltham, MA) and 1X P/S (Sigma-Aldrich, Saint Louis, MO) enriched with 20 ng/ml M-CSF (BioLegend, San Diego, CA) for 7-9 days to obtain M0. MDM cultures received a half medium change every 2-3 days^E1^. **CBMCs**: Mononuclear cells from umbilical cord blood (ICF obtained from mothers and study approved by Research Ethics Board, IWK Health centre, Halifax, Nova Scotia) were cultured as previously reported^E7^ at 1 x 10^6^cells/ml and passaged once per week for 4 weeks in StemSpan SFEM medium (Stem Cell Technologies, Vancouver, CAN) supplemented with 10 ng/ml rhIL-3 (Peprotech, Rocky Hill, NJ) (present only for the first week of culture), 10 ng/ml rhIL-6 (Peprotech, Rocky Hill, NJ) and 100 ng/ml rhSCF (Peprotech, Rocky Hill, NJ) in 5% CO2 at 37°C. At 5 weeks of culture, the medium was replaced by RPMI 1640 containing 10% FBS, 2 mM L-glutamine (GE Healthcare Life Sciences, South Logan, UT), 1X P/S, 50 µM 2-Mercaptoethanol (Sigma-Aldrich, Saint Louis, MO), 15 mM HEPES, 0.1 mM non-essential amino acids (GE Healthcare Life Sciences, South Logan, UT), 100 ng/mL rhSCF and 10 ng/mL rhIL-6. Mast cell purity (c-kit/CD117 expression at week 6) used in the experiments was ≥ 95%. CBMC cultures were devoid of dendritic cells and >99% positive for tryptase.

**Antibodies:** Following anti-human mAbs were purchased from BioLegend, San Diego, CA: CD3 (clone SK7, APC/CY7), CD11b (clone ICRF44, PE/CY7), CD86 (clone IT2.2, PE), CD163 (clone GHI/61, APC) CD206 (clone 15-2, APC/CY7). Anti-human mAb CD14 (clone 61D3, FITC) was purchased from Thermo Fisher Scientific, Waltham, MA. All respective mouse mAb ISO were purchased from BioLegend, San Diego, CA including, IgG1 (clone MOPC-21, APC/CY7), IgG1 (clone MOPC-21, FITC), IgG1 (clone MOPC-21, PE/CY7), IgG2b (clone MPC-11, PE) and IgG1 (clone MOPC-21, FITC), IgG1 (clone MOPC-21, APC). Anti-human mAb β2-AR (clone R11E1) was purchased from Bio-Rad Laboratories, Hercules, CA, USA. Mouse IgG1 ISO mAb (clone MG1-45) and anti-mouse IgG mAb (clone RMG1-1) were purchased from BioLegend, San Diego, CA. Anti-human IgE Fc mAb (clone B3102E8, HRP) was purchased from Southern Biotech, Birmingham, AL, anti-human αFCεRI mAb (clone AER-37, PE/CY7) from BioLegend, San Diego, CA and mouse IgG2b ISO mAb (clone eBMG2b, PE) used in CBMCs experiments from eBioscience, San Diego, CA.

*Polarization of MDM toward the M2a macrophage phenotype and treatment with epinephrine*
M0 were harvested using 2.5 mM EDTA (Sigma-Aldrich, Saint Louis, MO) in ice-cold PBS without Mg^2+^ and Ca^2+^ (GE Healthcare Life Sciences, South Logan, UT) and cultured in cRPMI with 20 ng/ml rhM-CSF, 20 ng/ml rhIL-4 and 20 ng/ml rhIL-13 (ImmunoTools, Friesoythe, Germany) for 72h to obtain M2a^E1^. Cell images were acquired using a Primo Vert Microscope (Carl Zeiss, Germany) and the ToupCam camera system (ToupTek, Zhejiang, China). 72h-polarized M2a were stimulated overnight (16h) with 1 µM EPI (Sigma-Aldrich, Saint Louis, MO) in TRIS buffer, pH 7.4 to assess cytokine production and surface marker expression and 48h-polarized M2a for 30 min or 2h to evaluate gene expression.
Quantitative kinetic chromogenic LAL assay showed absence of bacterial endotoxin in EPI sample (<0.0210 EU/ml of LPS).

*Cell staining and flow cytometry analysis*
M2a and M2a±EPI were detached using ice-cold 2.5 mM EDTA/PBS, washed twice with HBSS +3% FBS and incubated at 4°C for 30 min with multicolour mAB mixture (1:100 dilution) against CD14, CD11b, CD206, CD163, and CD86 or appropriate ISOs. For distinguishing dead cells from live cells, 2 drops of SYTOX^TM^ Green Ready Flow^TM^ Reagent (Thermo Fisher Scientific, Waltham, MA) were added directly on 1 x 10^6^ cells, incubated for 15 min at RT and analyzed in flow cytometry. The cells were analyzed using dual laser FACSCanto II equipped with FACSDiva Software (Becton Dickinson, Franklin Lakes, NJ, USA) and geometric MFI was calculated for each fluorochrome.

*Enzyme-linked immunoassay (ELISA)*
The presence of IL-10 (detection range 2-300 pg/ml), IL-6 (2-200 pg/ml), TNF (4-500 pg/ml), IL-12(p70) (4-500 pg/ml), IFN-γ (Thermo Fisher Scientific, Waltham, MA) and CCL-1 (detection range: 3.9-1000 pg/ml; R&D Systems, Minneapolis, MN, USA) in the supernatants was assessed by ELISA kits according to manufacturer’s instructions.

*RNA isolation and reverse transcription-quantitative PCR (RT-qPCR)*
48h-polarized M2a±EPI 30 min/2h were harvested (150 000 cells) using ice-cold 2.5 mM PBS/EDTA, washed once with HBSS, pelleted and lysed in Trizol (Sigma-Aldrich, Saint Louis, MO). RNA was isolated, using Direct-zol RNA MiniPrep column system (Zymo Research, Irvine, CA) and 200 ng RNA transcribed using iScript cDNA Synthesis Kit (BioRad, Hercules, CA) according to manufacturer's instructions. RT-qPCR was performed using HOT FIREPol® EvaGreen® qPCR Supermix (Solis BioDyne, Tartu, Estonia). RT-PCR analysis was performed using duplicates (and technical replicates of each biological replicate). Primers used: GAPDH forward: TTTTGCGTCGCCAGCCGAG and reversed: ACCAGAGTTAAAAGCAGCCCTGG; TNF forward: CAGGCGGTGCTTGTTCCTCA and reversed: GGCTTGTCACTCGGGGTTCG; IL6 forward: ATCCTCGACGGCATCTCAGC and reversed: TTCACCAGGCAAGTCTCCTCA; TGFB forward: GTTGTGCGGCAGTGGTTGAG and reversed: AGTGAACCCGTTGATGTCCACT; IL10^E8^ forward: GCCTAACATGCTTCGAGATC and reversed: TGATGTCTGGGTCTTGGTTC; CCL22 forward: TCCGTTACCGTCTGCCCCTG and reversed: GGCACTCTGGGATCGGCACA; IL1B^E9^ forward: CAGTGGCAATGAGGATGACTTG and reversed: AGTGGTGGTCGGAGATTCGT; CCL1 forward: TGTTGCTTCTCATTTGCGG and reversed: CTCTTTGCCTCTCTTCAGCTTG; LIGHT/TNFSF14 forward: CTCAGGCGTGTCTCTTCGGGTT and reversed: CACTCCTCATCCGTGGGTTGG; CCL18 forward: AAGCCCCAGCTCACTCTGACC and reversed: TGCCAGGAGGTATAGACGAGGC; CCL2^E10^ forward: CAGCCAGATGCAATCAATGCC and reversed: TGGAATCCTGAACCCACTTCT; ADRB2 forward: TAGAGATATGCAGAAGGAAGGGCAT and reversed: CCATGCAAAGAGGAACTGAACTGTA and IFNG forward: TGGCTTTTCAGCTCTGCATCGT and reversed: TTTTTCTGTCACTCTCCTCTTTCCA; ADRA2A forward: ACTGGACTACAAGGGCATGG and reversed: ACATCAAAACCAAGGCCAAG; ADRB1 forward: GTGGCCCTACGCGAGCAGAAG and reversed: GCGTAGCCCAGCCAGTTGAAGA; ADRB3 forward: CCCAATACCGCCAACACCAGT and reversed: CGGCCAGCGAAGTCACGA. GAPDH was selected as a reference gene, as described elsewhere^E11^. For transcriptional profiling of epinephrine-treated M2a macrophages for 30 min and 2h, ∆∆Cq levels were calculated after normalization with GAPDH and presented as a log2 fold change for M2a+epinephrine_30min_ treatment vs M2a and M2a+epinephrine_2h_ treatment vs M2a macrophages.

*Immunofluorescence staining of the β2-AR*
1.5x10^4^ M2a cells were left to adhere on 8-well chamber slide (µ-slide; Ibidi GmbH, Munich, Germany), washed twice with PBS and fixed using 4% paraformaldehyde (VWR International, Vienna, Austria) (30 min at RT). Cells were permeabilised with 0.2% Triton X-100 (Bio-Rad Laboratories, Hercules, CA) (2 min at RT), blocked with 10% FBS/PBS (30 min at RT) and incubated with a 1:25 dilution of mouse anti-human β2-AR 1°mAb (4 µg/ml) or mouse IgG1 ISO (1h). Anti-mouse IgG was used as a 2°Ab (2.5 µg/ml). DNA was visualized using DAPI (Bio-Rad Laboratories, Hercules, CA) (1:5000), slides mounted with fluoromount (Sigma-Aldrich, Saint Louis, MO) and analysed by fluorescence microscopy using Axio Observer Z1 inverted microscope with an AxioCam MRc5 camera and AxioVision software (Carl Zeiss Meditec, Jena, Germany). Relative quantification of β2-AR fluorescence intensity was performed using ImageJ software (version 1.52 e; National Institutes of Health, US).

*Stimulation of CBMC with supernatants from M2a-treated macrophages*
Two CBMC batches (1x10^6^ cells/ml) were pre-sensitized for 72h in complete RPMI with 2.5 µg/ml human myeloma IgE and 10 ng/ml rhIL-4 to upregulate FcεRI expression^E3^. Half of the medium was supplemented with 1 µM EPI or supernatants from M2a and EPI-treated M2a macrophages (n=3 PBMC donors) for 24h. β-hexosaminidase release assay was carried out by previously reported method^E12^.

$$\boldsymbol{\% release=}\frac{\left( \boldsymbol{O.D.}_{\boldsymbol{supernatant}}\boldsymbol{-}\boldsymbol{O.D.}_{\boldsymbol{HTB control}} \right)}{\left( \boldsymbol{O.D.}_{\boldsymbol{supernatant}}\boldsymbol{-}\boldsymbol{O.D.}_{\boldsymbol{HTB control}} \right)\boldsymbol{+}\left( \boldsymbol{O.D.}_{\boldsymbol{pellet}}\boldsymbol{-}\boldsymbol{O.D.}_{\boldsymbol{HTB control}} \right)}\boldsymbol{\times100}$$

*Statistical analysis*
The treatments M2a+EPI vs M2a and stimulation of CBMCs with macrophage supernatants were assessed using one-way analysis of variance (ANOVA) and Tukey's multiple comparison post-test. Differences between groups were considered significant at P value <0.05. Statistical analyses were performed with GraphPad Prism 6.0 for Macintosh (GraphPad Software, Inc., La Jolla, CA).

**List of abbreviations**

| BSA | bovine serum albumin |
| --- | --- |
| CBMCs | cord blood-derived mast cells |
| cRPMI | complete RPMI |
| DAPI | 4',6-diamidino-2-phenylindole |
| EDTA | ethylenediaminetetraacetic acid disodium salt dihydrate |
| EPI | epinephrine |
| FBS | fetal bovine serum |
| FSC-A | forward scatter area |
| FSC-H | forward scatter height |
| HBSS | Hank’s balanced salt solution |
| ICF | informed consent form |
| IL | interleukin |
| ISO | isotype control |
| mAb | monoclonal antibody |
| M-CSF | macrophage colony-stimulating factor |
| MDM | monocyte-derived macrophages |
| MFI | mean fluorescence intensity |
| PBMCs | peripheral blood mononuclear cells |
| PBS | phosphate buffered saline |
| rh | recombinant human |
| P/S | penicillin-streptomycin |
| RT | room temperature |
| SCF | stem cell factor |
| SSC-H | side scatter height |
| SSC-W | side scatter width |
| TRIS | tris(hydroxymethyl)aminomethane |
| β2-AR | β2-adrenergic receptor |

**References**

E1. Bianchini R, Roth-Walter F, Ohradanova-Repic A, Flicker S, Hufnagl K, Fischer MB, et al. IgG4 drives M2a macrophages to a regulatory M2b-like phenotype: potential implication in immune tolerance. Allergy. 2019;74(3):483-494

E2. https://www.stemexpress.com/blogs/peripheral-blood-mononuclear-cells

E3. Yamaguchi M, Sayama K, Yano K, Lantz CS, Noben-Trauth N, Ra C, et al. IgE enhances Fc epsilon receptor I expression and IgE-dependent release of histamine and lipid mediators from human umbilical cord blood-derived mast cells: synergistic effect of IL-4 and IgE on human mast cell Fc epsilon receptor I expression and mediator release. Journal of immunology (Baltimore, Md : 1950). 1999;162(9):5455-65

E4. Martinez FO, Sica A, Mantovani A, Locati M. Macrophage activation and polarization. Front Biosci. 2008;13:453-61

E5. Lu R, Pitha PM. Monocyte differentiation to macrophage requires interferon regulatory factor 7. J Biol Chem. 2001;276(48):45491-6

E6. Rey-Giraud F, Hafner M, Ries CH. In vitro generation of monocyte-derived macrophages under serum-free conditions improves their tumor promoting functions. PloS one. 2012;7(8):e42656

E7. Enoksson M, Ejendal KF, McAlpine S, Nilsson G, Lunderius-Andersson C. Human cord blood-derived mast cells are activated by the Nod1 agonist M-TriDAP to release pro-inflammatory cytokines and chemokines. J Innate Immun 2011;**3**(2):142-149

E8. Poole E, Avdic S, Hodkinson J, Jackson S, Wills M, Slobedman B, Sinclair J. Latency-associated viral interleukin-10 (IL-10) encoded by human cytomegalovirus modulates cellular IL-10 and CCL8 Secretion during latent infection through changes in the cellular microRNA hsa-miR-92a. J Virol. 2014;88(24):13947-55

E9. Yuan A, Hsiao YJ, Chen HY, Chen HW, Ho CC, Chen YY, Liu YC, Hong TH, Yu SL, Chen JJ, Yang PC. Opposite Effects of M1 and M2 Macrophage Subtypes on Lung Cancer Progression. Sci Rep. 2015: 24;5:14273

E10. Ormel PR, van Mierlo HC, Litjens M, Strien MEV, Hol EM, Kahn RS, de Witte LD. Characterization of macrophages from schizophrenia patients. NPJ Schizophr. 2017; 14;3(1):41

E11. Martinez FO, Sironi M, Vecchi A, Colotta F, Mantovani A, Locati M. IL-8 induces a specific transcriptional profile in human neutrophils: synergism with LPS for IL-1 production. Eur J Immunol. 2004 Aug;34(8):2286-92.

E12. Kuehn HS, Radinger M, Gilfillan AM. Measuring mast cell mediator release. Curr Protoc Immunol. 2010;Chapter 7:Unit7.38

**Supplementary figure legend**

**
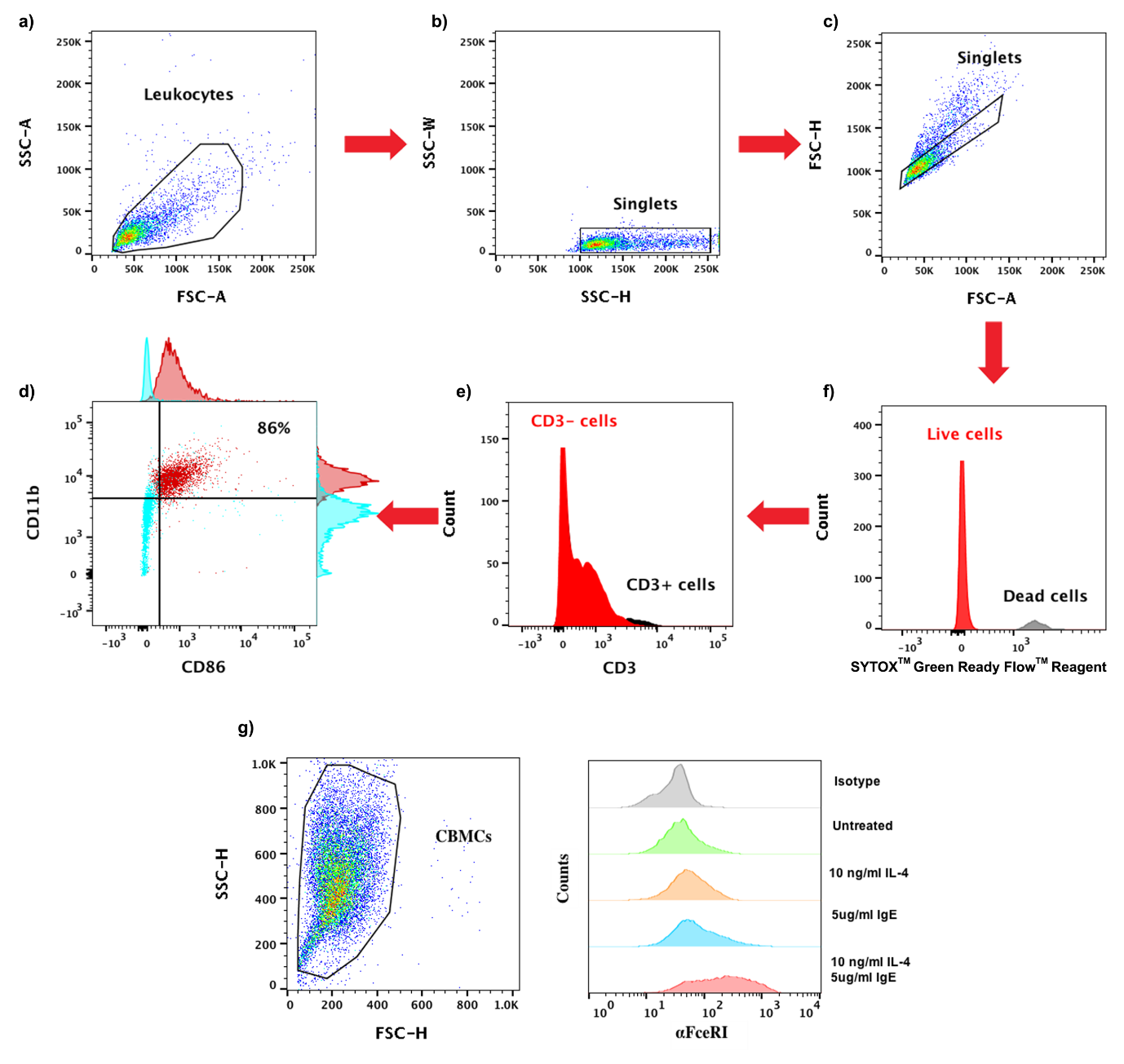
**

**Figure S1**. Flow cytometry analysis representing MDM purity (a-f) and FcεRI expression on CBMCs (g). Assessment of adherent monocyte-derived macrophages after 72h in M-CSF differentiation medium by flow cytometry by gating leukocytes (a), single cells SSC-W and SSC-H (b) and FSC-H and FSC-A (c). SYTOX™ Green Ready Flow™ Reagent was used to gate for live cells (red label) (d). The percentage of live cells was 87±2%. Live cells were gated using anti-CD3 (e) and CD3^-^ cells (red, 98%) were further analysed using anti-CD11b and anti-CD86 (f). Isotype control is represented in cyan. CBMCs were cultured for 4 days in the presence of rhIL-4 and human myeloma IgE and stained with monoclonal antibody against FcεRI before or after stimulation with rhIL-4 myeloma IgE or rhIL-4 and IgE (g).


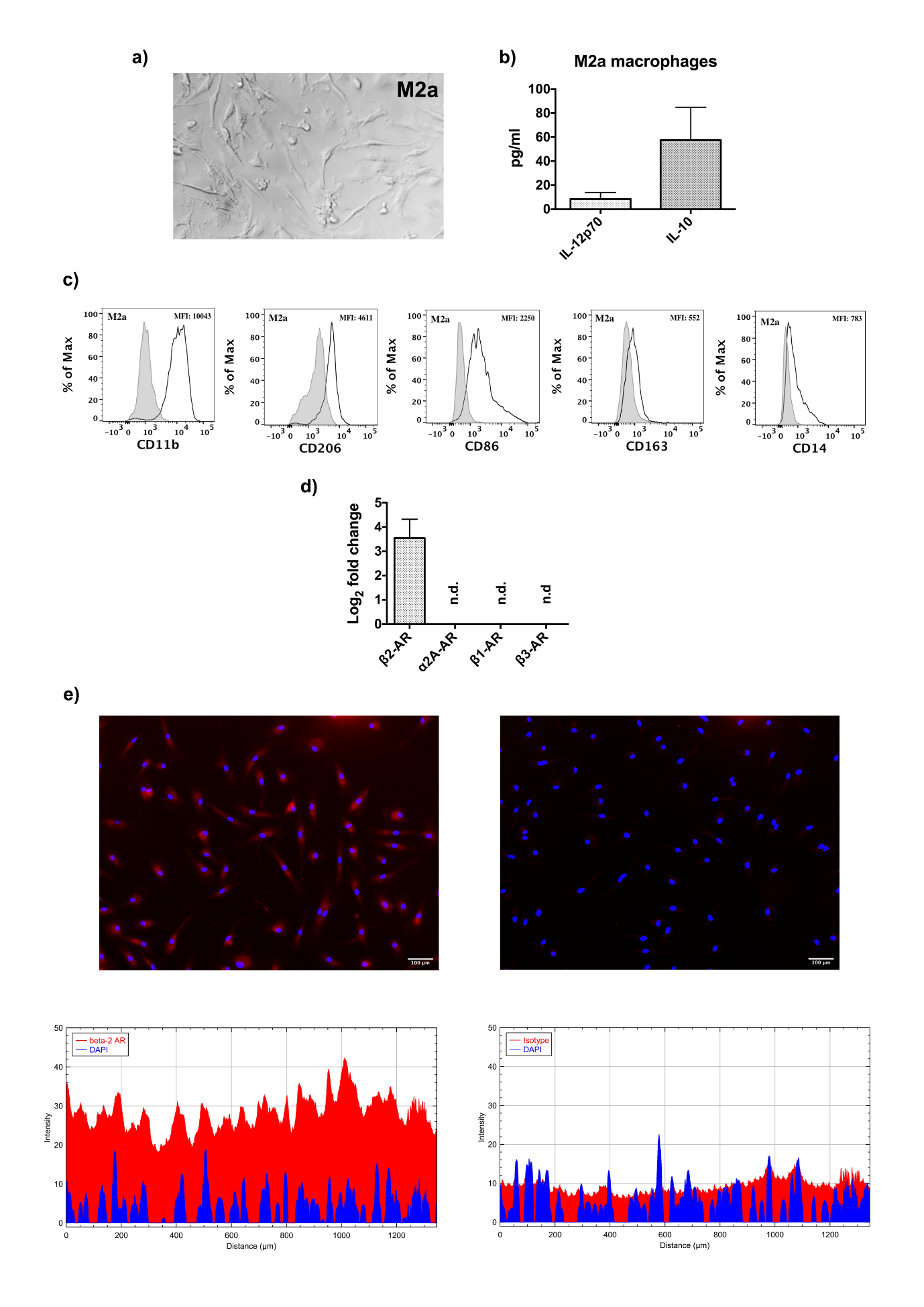


**Figure S2**. Phenotypic characterization of M2a macrophages and the β2-adrenergic receptor expression. M2a macrophages were obtained after treatment with rhM-CSF for 9 days and further stimulation with rhIL-4 and rhIL-13 for 72h and characterized phenotypically in light microscopy (a). IL-12 and IL-10 cytokine production was assessed by ELISA (b) and surface marker expression in flow cytometry (c) (representative image of n=6 donors). The shaded gray histogram represents the relevant isotype control antibodies. RT-PCR revealed the presence of β2-AR mRNA (mean±SD, n=4 normalized to Caco-2 cell line used as a negative control) (d) and β2-AR protein expression in M2a macrophages detected by immunofluorescence (representative confocal microscopy image (n=3) (e). β2-AR expression was quantified using ImageJ software. The red peaks represent fluorescence intensity of M2a macrophages stained with the β2-AR antibody or respective isotype control.

**
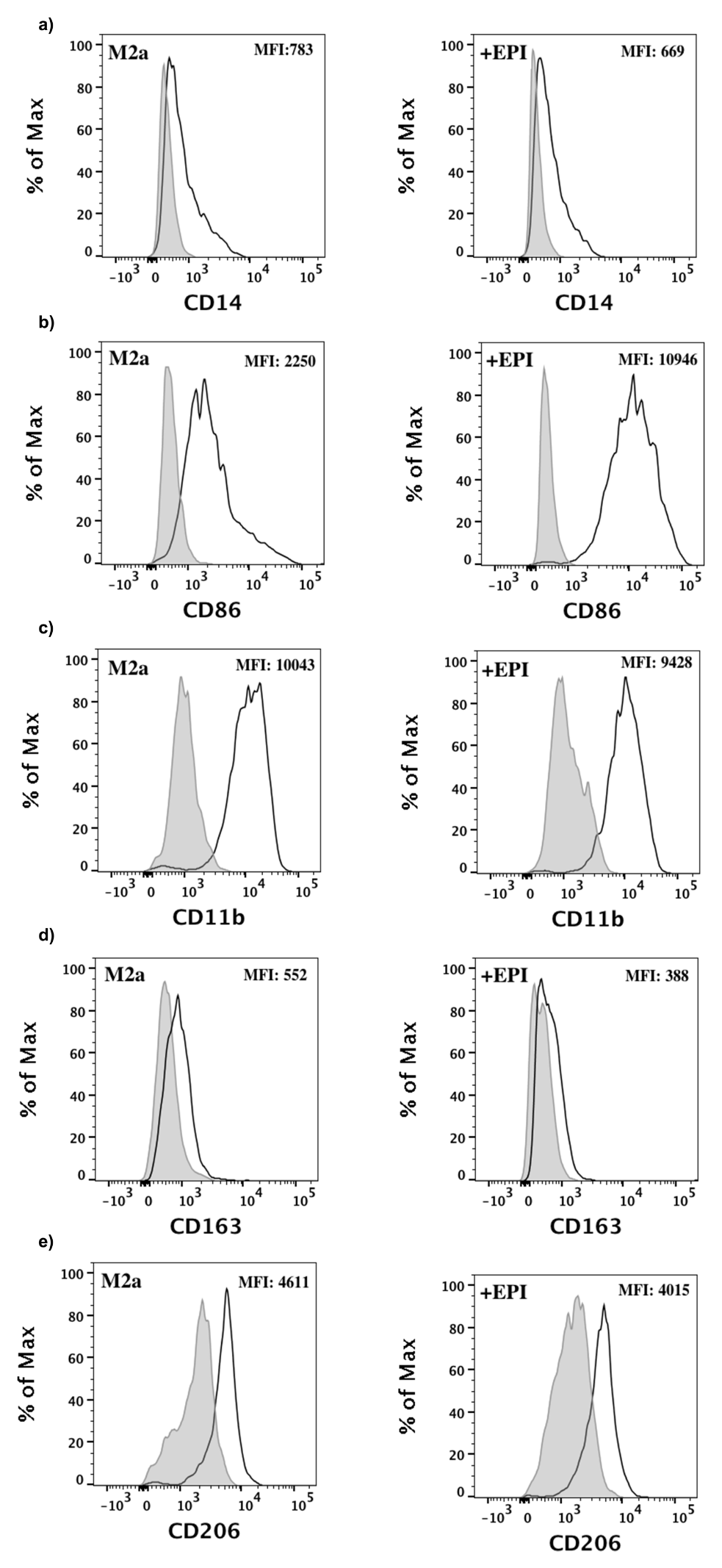
**

**Figure S3**. Flow cytometry analysis of surface marker expression in the presence and absence of epinephrine. M2a macrophages were incubated overnight (16h) with 1 µM epinephrine (EPI) and surface marker expression including CD11b (a), CD206 (b), CD86 (c), CD163 (d), and CD14 (e) (representative image of six independent donors) was assessed on M2a macrophages or EPI-treated M2a. The shaded gray histogram represents the relevant isotype control antibodies.
